# An Update from the Editorial Board of Nutrients

**DOI:** 10.3390/nu7075236

**Published:** 2015-07-08

**Authors:** Jonathan Buckley, Peter Howe

**Affiliations:** 1 Nutritional Physiology Research Centre, Sansom Institute for Health Research, University of South Australia, GPO Box 2471, Adelaide, South Australia 5001, Australia; 2 Clinical Nutrition Research Centre, University of Newcastle, Callaghan, NSW 2308, Australia

The Editorial Board of Nutrients is pleased to announce that the journal impact factor has continued to rise to reach 3.270 in 2015. This impact factor means that Nutrients is now ranked 21st out of 77 journals published in the field of Nutrition and Dietetics, and is the second ranked Open Access journal in this field. Given this is only the fifth year since Nutrients was added to scientific indexing databases, this high ranking is testament to the considerable efforts of the Editorial Board.

As Nutrients’ impact factor has risen, so has the number of submissions received, with almost 600 submissions received in 2013, more than 700 in 2014, and 640 received already in the first half of 2015. However, the rejection rate has also increased over this same period, such that almost 55% of submissions are rejected. This relatively high rejection rate reflects the considered decision by the Editors to accept only high quality manuscripts for publication.

Dealing with the increasing number of submissions and publications has presented some significant challenges for the Editorial Board, but despite this, the average time from submission of manuscripts to publication has decreased progressively from 95 days in 2013, to 86 days in 2014 and 69 days for the first half of 2015. This reduction in time taken to review and publish manuscripts that are increasingly impactful assists substantially in ensuring rapid progression in knowledge advancement within the field of Nutrition and Dietetic research.

While the Editorial Board holds full responsibility for the editorial processes and decisions related to the acceptance, or otherwise, of submitted manuscripts, they are well supported by the administrative activities of the MPDI team, which has also contributed to the ongoing success of the journal. Part of the success of Nutrients is in no small part due to the willingness of MDPI to give full responsibility to the Editors for overseeing all aspects of the editorial process, and adapting their back-room activities to best support the strategies and decisions of the Editors.

The Editors-in-Chief are very grateful to our Editorial Board members and to the staff of MDPI for all of their efforts that have contributed to the ongoing success of Nutrients, and look forward to Nutrients continuing to increase its impact within the field into the future.

